# Historical Biogeography of Five *Characidium* Fish Species: Dispersal from the Amazon Paleobasin to Southeastern South America

**DOI:** 10.1371/journal.pone.0164902

**Published:** 2016-10-14

**Authors:** Daniel Poveda-Martínez, Chrystian C. Sosa, Katherine Chacón-Vargas, Víctor Hugo García-Merchán

**Affiliations:** 1 Grupo de Evolución, Ecología y Conservación EECO, Universidad del Quindío, Armenia, Colombia; 2 Grupo de investigación y asesoría en estadística, Universidad del Quindío, Armenia, Colombia; 3 International Center for Tropical Agriculture (CIAT), Km 17 recta Cali-Palmira, Cali, Colombia; Universitat Trier, GERMANY

## Abstract

*Characidium* is a Neotropical fish genus. Its distribution ranges from eastern Panama to northern Argentina, and it is an important component of the Neotropical ichthyofauna present in the major rivers of South America. We here provide an approximation to the dispersal and historical distributions of *Characidium*. The biogeographic history of five species of the genus was analyzed through nuclear *RAG-2* and mitochondrial *16S* genes and a time-calibrated phylogenetic analysis using three outgroup species. A biogeographical reconstruction was performed to estimate ancestral geographic ranges and infer the historical events that impacted the geographic distributions of *Characidium* species. Our results showed *Characidium* as a monophyletic group. The molecular clock suggests that the most recent common ancestor of *Characidium* originated during the Eocene, about 50.2 Mya. In addition, different dispersion and vicariance events could be inferred, which possibly gave rise to the present geographical distribution of the genus. Our results point to the rise of the Andean mountains and sea fluctuations as being important events in the formations and delimitation of different rivers, which influenced the distribution of South American ichthyofauna.

## Introduction

The biogeographic history of freshwater fishes in South America is still challenging to biologists due to its complex hydrographic zone, which has originated a great diversity of fishes (i.e. Amazonas basin) making the freshwater fishes the major group of vertebrates on the planet. The freshwater fishes are a study model which provides an opportunity to better understand evolutionary patterns and diversification processes in South American basins [1]. The study of freshwater species has two main advantages 1) organisms have evolved confined to different river basins throughout the continent and 2) the restriction of their current distribution is primarily due to river basin rearrangements during geological history [2]. These characteristics make possible to understand the evolutionary dynamics of freshwater species trough river [1, 2].

*Characidium* Reinhardt, 1867, is a neotropical fish genus of the Crenuchidae family [3–4]. Species of this family are small in sizes, ranging from 30 to 80 mm standard length. Crenuchidae range from East Panama to Northeastern Argentina in the east and west flanks of the Andes [3–7]. They are usually found in lentic ecosystems, small streams, lowland rain forests and swamps of coastal plains [3, 8]. They are also found at different altitudes and environments under diverse climatic and topographic conditions [4, 6–8].

Previous morphological studies suggested that *Characidium* is a monophyletic group based on one synapomorphy: a black spot near the base of the middle caudal-fin ray, usually formed by a discrete cluster of chromatophores restricted to the caudal fin rays [9, 10]. In spite of the phylogenetic relationships of *Characidium* based on morphological traits [5, 9, 10], the phylogeny relationships of this genus is disjointed because there is not a molecular consensus among studied species [11, 12]. In addition, previous studies on geographical distribution at limited spatial scales had reduced power to reconstruct the biogeographical history of *Characidium*.

Based on the combined approximation of molecular phylogenetics and historical biogeography, the objective of this study was to identify the evolutionary processes that shaped the current geographic distribution of five species of *Characidium* in the main river basins of South America.

## Material and Methods

### Molecular information

For the phylogenetic reconstruction of *Characidium* species we used nuclear gene *RAG2* and the mitochondrial gene *16S*, one sequence per gene per species, previously analyzed by Calcagnotto *et al*. [11] and Oliveira *et al*. [12]. Sequences were downloaded from GenBank (http://www.ncbi.nlm.nih.gov/genbank/)). Five species were used in this analysis, based on the current molecular information for both genes: *Characidium fasciatum*, *C*. *purpuratum*, *C*. *vidali*, *C*. *laterale* and *C*. *pterostictum*. *Crenuchus spilurus*, *Poecilocharax weitzmani* (Crenuchidae: Crenuchinae), *Hoplias* sp. (Erythrinidae) were used as outgroups based on the possession of more inclusive synapomorphies shared with the ingroup *Characidium* [5, 12, 13]. In addition, we confirmed the origin of the species with the data from Calcagnotto *et al*. [11] and Oliveira *et al*. [12] using the fishes catalog of California Academy of Science (http://www.calacademy.org/), Museu Nacional Universidad Federal do Rio de Janeiro (http://www.museunacional.ufrj.br/) and the Check List of fishes of Central and South America [14] (S1 Table).

### Alignment and sequence analysis

Multiple sequence alignments were constructed for each gene using the MUSCLE algorithm implemented in MEGA v.6.0.6 [15]. Then, poorly aligned positions and divergent regions of the alignment were eliminated with GBLOCKS v.0.91b [16]. Finally, the saturation substitution index (I_ss_) of each sequence was estimated using the Xia approach [17] to evaluate the occurrence of substitution saturation, and the proportions of conserved and polymorphic sites. Likewise, the transition/transversion rate was computed using DAMBE v.5.5.1 [18].

### Bayesian inference and divergence times

A phylogenetic reconstruction was performed using BEAST v.1.7.5 [19]. We used the General Time Reversible + Gamma parameter + invariants sites (GTR+Γ+I) nucleotide substitution model for the sequences of each gene as selected through Jmodeltest2 v.2.1.3 [20]. Yule speciation process and a relaxed molecular clock were used in BEAST. Due to the absence of an appropriate fossil record, we used a calibration time point according to Warnock *et al*. [21] in order to calibrate the BEAST runs. The calibration time corresponds to the middle Miocene (between 12.5 and 5 Mya), period in which multiple hydrogeological rearrangements took place in Brazilian coastal basins [22, 23] and where a vicariance event could have played a role in the divergence of *C*. *vidali* and *C*. *pterostictum* in this region. Other studies have reported the isolation and disjunctive distribution for other freshwater fishes on the Brazilian coastal basins [3, 24–26]. In Addition, a normal distribution in the calibration point was used as in Warnock *et al*. [21]. Finally, MCMC chains were ran for 10 million generations and sampled every 1000th generation. Stability and sufficient mixing of parameters (ESS>200) was checked using Tracer v.1.6 [27], then a consensus tree was generated using TreeAnnotator v.1.7.5 [19] and visualized on FigTree v.1.3.1 [28].

### Species distributions on the study areas

Occurrence records (points of presence) of the eight species used in the phylogenetic analysis were obtained from online databases and other organizations such as: Global Biodiversity Information Facility (http://www.gbif.org/), Smithsonian Tropical Research Institute (http://www.stri.si.edu/), Fishnet (http://www.fishnet2.net/), California Academy of Science, American Museum of Natural History (http://www.amnh.org/), and Biodiversity Information Projects of the World (http://www.tdwg.org/). Missing coordinates were georeferenced via Google Earth v.7.1.2 [29].

**Fig 1:**
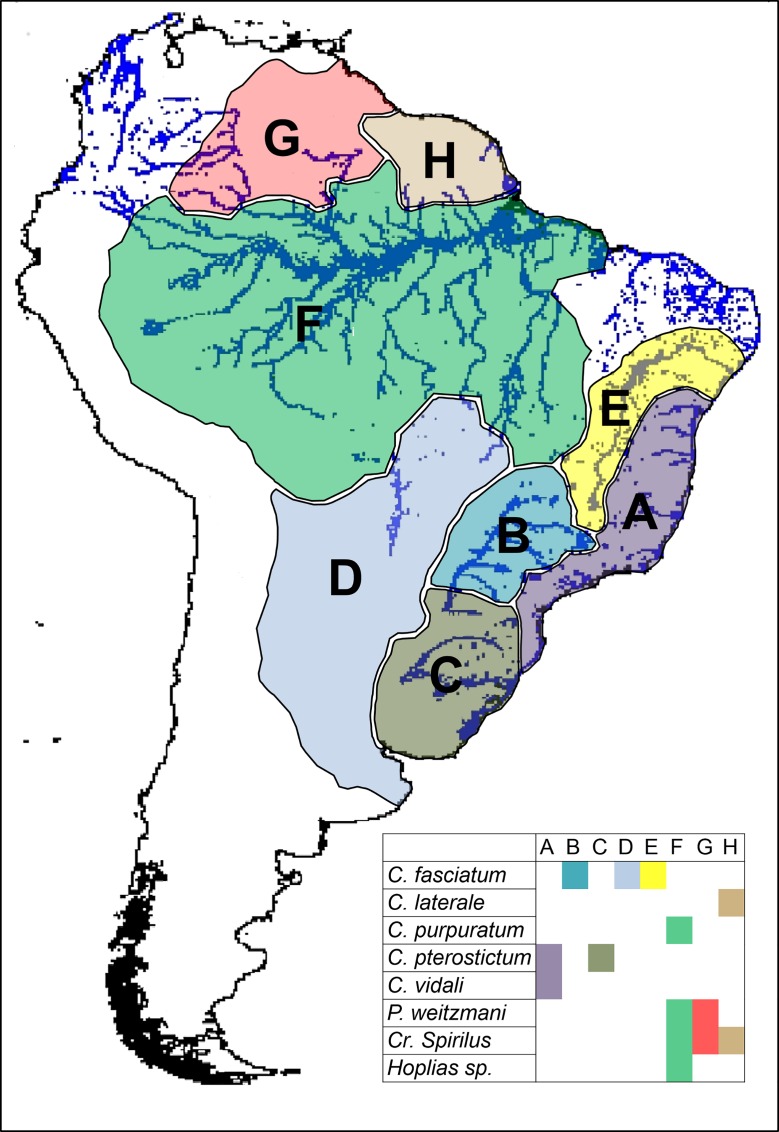
Ichthyografical region and distribution of five species of *Characidium*. Icthyogeografical region according to Vari and Malabarba [31] and Chiachio *et al*. [32] are A: Atlantic Coastal Drainage, B: Upper Parana basin, C: Uruguay basin and Coastal Drainage, D: Paraguay basin and Lower Parana, E: São Francisco basin and Drainages of Northeastern of Brazil, F: Amazon basin, G: Orinoco basin, and H: Coastal Drainage of Guyana. Blue lines show the principal waterbody of South America. The square shows the current distribution of the taxa considerer in this study.

Finally, DIVA-GIS v.7.5.0 [30] was used to assign biogeographical areas according to Vari and Malabarba [31] and Chiachio *et al*. [32]. The ichthyographical regions are A: Atlantic Coastal Drainage, B: Upper Parana Basin, C: Uruguay Basin and Coastal Drainage, D: Paraguay Basin and Lower Parana, E: São Francisco Basin and Drainages of Northeastern of Brazil, F: Amazon Basin, G: Orinoco Basin, and H: Coastal Drainage of Guyana (Fig 1).

### Historical Biogeography Reconstruction

Two approaches were implemented: a maximum-likelihood analysis of biogeographic history [33, 34] using a dispersal-extinction-cladogenesis (DEC) model of geographic range evolution and a Statistical Dispersal-Vicariance Analysis (S-DIVA; [35]). The DEC model specifies instantaneous transition rates between discrete distribution areas along the branches of a phylogenetic tree, and uses these rates to assess likelihoods of ancestral distributions at cladogenetic events. This method obtains its parameters from a topology tree in an ultrametric chronogram format, a global extinction and a dispersal rate averaged across the tree, and the probability of connectivity among areas through time. Also, DEC method gives equal weight to dispersal and vicariance *a priori*. S-DIVA is a statistical application of DIVA [36], which is a parsimony method of historical biogeography. S-DIVA was used to obtain trees with the probabilities of ancestral areas [37] or clades with unknown sister groups [38, 39]. S-DIVA inputs bifurcated trees and a list of plausible adjacent areas. S-DIVA is recommend if there is some *a priori* knowledge of vicariance events; in this case, vicariance could have played an important role in the biogeographic history of the evaluated group of *Characidium*. We used 10.000 trees from Bayesian analysis, the consensus tree form the TreeAnotator and the distribution of the species through all biogeographical areas to perform DEC and S-DIVA analyses in RASP v.3.0 [40].

## Results

### Sequence analysis

Sequence alignments of 1482bp total length were obtained and analyzed. A total of 1141bp (76.99%) were conserved positions and 341bp (23.01%) were polymorphic sites. Saturation substitution analysis showed that, according to the comparison proposed by Xia *et al*. [17] between the I_ss_ and the I_ss_ complete (I_ss.c_), no saturation of phylogenetic signal was observed (S2 Table).

### Phylogenetic inference and operational areas

**Fig 2:**
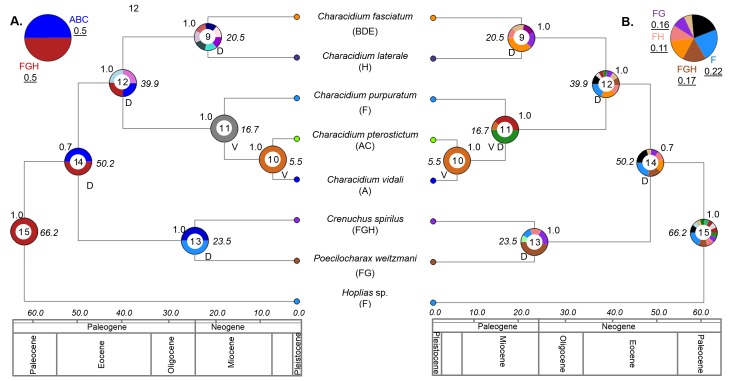
Time-calibrated phylogeny from BEAST analysis and reconstructed ancestral distributions from RASP. This showing the interrelationship among species of *Characidium* and their divergence times. Numbers below branches are posterior probabilities obtained in the Bayesian Inference analysis. Each area is marked in colors that are seen in the pie charts node indicating the likelihood of occurrence in an ancient area (the codifications of these areas are in S1 Fig). On top of the pie charts the posterior probability of each tree node. The divergence time values (Mya) are in italics on the left side of the pie. A and B show the detail diagram of the center of origin of *Characidium* ancestor of the clade and their probabilities underlined. V and D represent vicariance or dispersion in each node.

The phylogenetic tree suggests a common origin for the five *Characidium* species studied (Posterior Probability-PP = 0.7) (Fig 2). In addition, the tree shows a close relationship between *C*. *vidali* and *C*. *pterostictum* (PP = 1). Both taxa are present in the Atlantic coastal drainages of Brazil (Region A) and *C*. *pterostictum* is also distributed in Uruguay-Paraná basin (Region C). *C*. *purpuratum* was grouped as sister species of this clade (PP = 1) (Fig 2) and has been reported in the drainages of the Andean in the Amazon River margin in Ecuador and Bolivia (Region F) (Fig 1). *Characidium laterale* is distributed in the Potaro and Mazaruni Rivers in Guyana (Region H). This taxon shows a close relationship with *C*. *fasciatum* (PP = 0.997) (Fig 2) which is widespread in northern tributaries of São Francisco River drainage (Region E) and Paraná River basin (Region B, D) (Fig 1). Outgroups *Crenuchus spilurus* and *Poecilocharax weitzmani* clustered close to *Characidium*, while *Hoplias* sp. was the most distant taxon (Fig 2). Furthermore, *P*. *weitzmani* and *C*. *spilurus* were distributed in the Orinoco and Amazon River basin (Regions G and F, respectively). *C*. *spilurus* is present in the coastal rivers in Guyana (Region H), and *Hoplias* sp. occurs in the Amazon Basin (Region F) (Fig 1) (S1 Table). This supports the Amazon basin as ancestral area for *Characidium*.

### Divergence times and biogeographical reconstruction

The results from the BEAST analysis suggest that the ancestor of *Characidium* genus was originated during the Eocene, approximately 50.2 Mya (Fig 2: node 14). Both analytical methods suggest the clade was originated in a wide area including the Amazon Paleobasin (Regions F, G, H) (Fig 1). The probability (prob) of F, G, H was 0.5 in S-DIVA and 0.66 in DEC (in this last one we include all the probabilities that had F, G, H regions) (Fig 2A and 2B; S1 Fig). There was high concordance using DEC, suggesting that the origin of *Characidium* was in an ancestral region which included the Amazon region (prob(F) = 0.22; prob(FH) = 0.11; prob(FG) = 0.16; prob(FGH) = 0.17). Nevertheless, S-DIVA also predicts a possible origin in a wide area of the Brazilian shield and the Brazilian Atlantic Coastal watersheds (prob(ABC) = 0.5) (Fig 2A).

Within the genus, three clades were identified (Fig 2; nodes = 9, 10, 11). This separation took place at the end of the Eocene between 38.9 and 40.9 Mya (mean = 39.9 Mya). The distribution of ancestor of clades is best explained assuming five dispersal events (expansion of distribution range) that originated in the Amazon basin (Fig 2, prob = 0.19) in direction to Guyanese rivers, the Upper and Lower Parana, drainages of Northeastern of Brazil and lastly, Atlantic coastal drainages. The clade consisting of *C*. *fasciatum* and *C*. *laterale* could have originated at the end of the Oligocene or beginning of the Miocene between 31.3 and 9.8 Mya (mean 20.4 Mya) from two dispersal events on their ancestor. During this period the Amazon Paleobasin was connected with drainages of the Upper Parana (region BE-FGH; prob = 0.38 in DEC; prob = 0.14 in S-DIVA) (Fig 2, S1 Fig). Separate dispersions going directly into the basins of the Amazon and São Francisco rivers in Brazil and some other rivers from Guyana region were supported with a lower probability (p = 0.12) but may have an important role in explaining these dispersals events.

The second clade identified in the phylogenetic analysis (Fig 2; node = 11) could have been originated in the Miocene, between 28.6 and 7.5 Mya. Each of these biogeographical events occurred from the western limits of the Amazon to the Upper Parana basin, basins of Uruguay and drains on the Atlantic coast in Brazil. In addition, *C*. *vidali* and *C*. *pterostictum* are the latest clades in this study and could have originated in the Pliocene about 5.5 Mya (between 11.3 y 1.5 Mya). DEC and S-DIVA analyses were consistent in predicting that the vicariance events gave rise to the divergence of this clade between drainages in the Atlantic Coast and Upper Parana (Figs 1 and 2). The current distribution of the five species of *Characidium* was likely generated from successive dispersal events in the most ancestral nodes in the Amazon, and only two vicariance events could have played an important role in the speciation of the most recent clades in Atlantic coastal drainages.

## Discussion

### Monophyly of *Characidium*

In this study, the monophyly of five species of *Characidium* was corroborated using two molecular markers. Our results were consistent with the phylogenetic hypothesis exposed by Buckup [5, 9] using other species and based on morphological characters. However, our approach does not allow to conclude that the whole genus is monophyletic due to the large number of species (close to 58) that it includes [3, 4].

### Divergence times and biogeographical reconstruction

#### Geological changes in the Eocene

Molecular phylogenetics and phylogeography-based methods help reconstructing historical patterns that led to the current geographical distributions [41–43], and freshwater fishes represent an excellent model to carry out these type of studies [1]. This analysis showed that the most recent common ancestor (MRCA) of the five species of *Characidium* was originated in the Eocene about 50.2 Mya. This period contained many geological events that could have favored the diversification of the South American ichthyofauna [1, 44–46]. Those regional rearrangements within the aquatic ecosystems facilitated the adaptive radiation of many groups of taxa during the Cretaceous-Paleogene limit [44, 47–48]. During this period, a prolonged series of marine transgressions and regressions, active tectonic activity in the Andes and in the east coast of Brazil [25], likewise progressive global cooling from late Eocene were central to the origin of the genus and other Neotropical groups as Characoidea, Loricarioidea and Erythrinoidea [45]. Many studies have oversimplified the age and cause of diversification of Neotropical ichthyofauna by ascribing the formation of the Amazon region to the final uplift of the Andes (11 Mya) [46]. Nevertheless, this proposition does not take into account the most ancient history of the ichthyofauna, the development of the Andes in the past 90 Mya and the immense watersheds in the lowlands that have existed at least since 67 Mya [1, 44–46].

#### Amazon Paleobasin as center of origin for five *Characidium* species

This study confirmed the Amazon Paleobasin as the center of origin of the most recent common ancestor of five species of *Characidium* (Fig 2A and 2B). This region was strongly influenced by geological forces and has been reported as the center of origin of most of the Neotropical biodiversity [32, 49–52]. An alternative hypothesis for the biogeographic history of the five *Characidium* species suggested by S-DIVA, shows a possible origin of the ancestor of this clade in the Atlantic Coastal Drainage. This zone has a complex and ancient geological history dating back to the final separation of Africa and South America about 100 Mya [53, 54]. Coastal Drainages of Southeastern Brazil are important areas where some families of fishes originated [22, 23]. The ichthyofauna of coastal drainages from East Brazil has a great biogeographical importance, especially because of its high degree of endemism [3, 25–26, 53–54].

The distribution of *C*. *fasciatum* in the São Francisco basin could have occurred through of a dispersal event on their ancestor between this river and the Amazon Paleobasin [22]. A similar event was reported in the ancestor of three species of the Loricariidae family like Curimatidae family species, which has a broad distribution in São Francisco basin and the Amazon basin [22, 55]. This led to the hypothesis that São Francisco basin constitutes a hybridization area because some components of the ichthyofauna that are represented in this basin are closely related with species in the Amazon basin [55, 56]. Given that *C*. *fasciatum* is present in São Francisco basin and the Parana River basin, both main basins of South America, we suggest a possible hypothesis of a founder effect. Probably, this species has led to the formation of new species on other South American drainages. This aspect is concordant with the divergent time of *C*. *fasciatum* as an ancestral species [5] and its ancestral forms that tends to be confused with another species as *C*. *zebra* in other drainages of South America.

The current distribution of *C*. *laterale* in the Potaro and Mazurini rivers (tributaries of the Essequibo basin) (Fig 1) was probably due to at least to two dispersal events of its ancestor of this taxon. The dispersive route between the Guyanese rivers matches with the existence of an exchange of ichthyofauna between the Atlantic Coastal Drainages of the Eastern Guianas (Guyana, Suriname, and French Guiana) and the Eastern portion of Amazon Paleobasin [57]. This exchange took place through a marine coastal corridor where the salinity was reduced by the discharge of the Amazon basin and other coastal drainages confluence of rivers, accompanied by short periods of marine regression that occurred in the Pleistocene and / or the formation of alluvial fans and river capture [55]. This coastal corridor apparently covered an area from the mouth of the Essequibo basin to the mouth of the Amazon River.

The dispersion event informed on the ancestor of *C*. *purpuratum* (Amazon region) could have shaped its current distribution. Although *C*. *purpuratum* is not the most representative specie of the Amazon basin, the rise of the Andes, played an important role in the formation of the modern Amazon basin [46, 58], also influenced in the redistribution and reorganization of different taxa along the Amazon and its margins. This taxon is confined to the western margin of the Amazon basin, on the border with the Andean uplift, which is a feasible hypothesis based on the age of divergence (16 Mya). Due to the above, the ancestor of *C*. *purpuratum* could not have migrated through this geographical barrier, confining this taxon to have a Cis-Andean distribution because of the current limits of South American basins, as well as the wildlife found there which was largely established because of the uplift of the Andes over the past 20 Mya [58]. Moreover, it is possible that other species of *Characidium*, for example, *C*. *caucanum*, *C*. *phoxocephalum* that reached a Trans-Andean distribution [7], and *C*. *marshi* that colonizes some tributaries of the Tuira river basin at the East of Panama [5] dispersed before the maximum orographic elevations took place. The implementation of an analysis with species with trans-Andean and trans-isthmus distributions would be essential to fully understanding of the *Characidium* biogeography.

The last clade, *C*. *pterostictum* and *C*. *vidali*, diverged about 5.5 Mya in the Pleistocene from a vicariance event between Parana River basin and Atlantic Coastal Drainage. Changes in sea levels in the Pleistocene (5 Mya) could have isolated many of the coastal drainages and Atlantic tributaries of the Upper Parana, and consequently could have generated several allopatric speciation events through vicariance in different groups of strictly freshwater fishes [26, 44, 59, 60]. Once the sea retreated, new species may have dispersed throughout the Parana basin and Coastal Atlantic Drainages. Leitão and Buckup [3] documented at least seven *Characidium* taxa distributed in the Atlantic coastal drainage and would show that these coastal rivers have played a major role as a refuge for species during periods of marine transgressions and regressions as is the case of *C*. *vidali* during the Pleistocene [3, 25]. To our knowledge, this is the first study that provides an approximation to the biogeographical history of *Characidium*. Our study identifies the Amazon Paleobasin as the origin center of five species of this genus of freshwater fish. Incorporating a greater number of species distributed in this region and an analysis using multiple molecular markers in combination with morphological data and morphometric techniques is recommended for a better resolution of the evolutionary history of *Characidium*.

## Supporting Information

S1 Fig Codifications of ancestral areas. (TIF)

S1 Table Information about species included in the study. (DOCX)

S2 Table Nucleotide substitution models and Substitution Saturation rate. Nucleotide substitution models, Substitution Saturation using the index of substitution saturation (I_ss_) and the transition/tranversion rate estimated for each gene.
(DOCX)
