# Supplementary figures and images for: Historical Biogeography of Five Characidium Fish Species: Dispersal from the Amazon Paleobasin to Southeastern South America
Source: PLoS One. 2016 Oct 14;11(10):e0164902. doi: 10.1371/journal.pone.0164902 (PMC5065214; doi:10.1371/journal.pone.0164902)

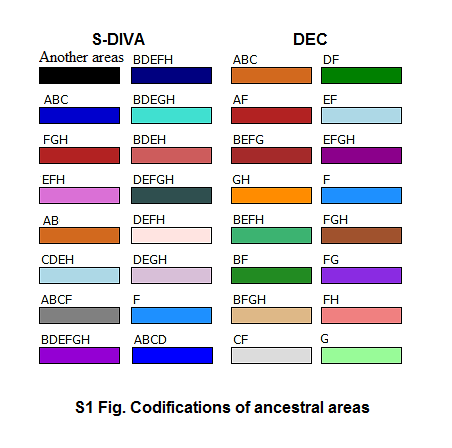

Supplement: S1 Fig — (TIF) [file pone.0164902.s001.tif]
