# Supplementary material for: Historical Biogeography of Five Characidium Fish Species: Dispersal from the Amazon Paleobasin to Southeastern South America
Source: PLoS One. 2016 Oct 14;11(10):e0164902. doi: 10.1371/journal.pone.0164902 (PMC5065214; doi:10.1371/journal.pone.0164902)
Supplement: S1 Table — (DOCX) [file pone.0164902.s002.docx]

Supplementary Table 1. Species included in the study. MNRJ: Museu Nacional da Universidade Federal do Rio de Janeiro; LBP: Laboratório de Biologia e Genética de Peixes, Universidade Estadual Paulista; AMNH: American Museum of Natural History.

| **Number** | **Nº collection** | **GenBank voucher (*16S/RAG2*)** | **Species** | **Distribution area** | **Reference** |
| --- | --- | --- | --- | --- | --- |
| **1** | MNRJ 12838 | AY788003/ AY804064 | *Characidium vidali* | A | [11] |
| **2** | LBP 2132-21388 | HQ171284/ HQ289381 | *Characidium pterostictum* | B/C | [12] |
| **3** | LBP 7614-36938 | HQ171398/ HQ289491 | *Characidium laterale* | D/H | [12] |
| **4** | AMNH 233314 | AY787988/ AY804052 | *Characidium purpuratum* | F | [11] |
| **5** | AMNH233251 | AY787992/ AY804054 | *Characidium fasciatum* | B//E/F/G/H | [11] |
| **6** | LBP6907-33264 | HQ171377/ HQ289471 | *Crenuchus spilurus* | F/G/H | [12] |
| **7** | LBP7078-40500 | HQ171414/ HQ289507 | *Poecilocharax weitzmani* | F/G | [12] |
| **8** | - | AY788031/ AY804087 | *Hoplias* sp. | F | [11] |
