# Supplementary material for: Historical Biogeography of Five Characidium Fish Species: Dispersal from the Amazon Paleobasin to Southeastern South America
Source: PLoS One. 2016 Oct 14;11(10):e0164902. doi: 10.1371/journal.pone.0164902 (PMC5065214; doi:10.1371/journal.pone.0164902)
Supplement: S2 Table — Nucleotide substitution models, Substitution Saturation using the index of substitution saturation (Iss) and the transition/tranversion rate estimated for each gene. (DOCX) [file pone.0164902.s003.docx]

Supplementary Table 2. Substitution Saturation estimated for each gene using the index of substitution saturation (Iss) [17] and rate of transitions and transversions evaluated in DAMBE v.5.5.1 [18].

| **Molecular Marker** | **Evolutionary model** | **Transitions** | | **Transversions** | | **Iss < or > Iss.c** |
| --- | --- | --- | --- | --- | --- | --- |
| *16S* | GTR+Γ+I | | 0,184 | | 0,086 | 0,1096 < 0,6 |
| *RAG2* | GTR+Γ+I | | 0,215 | | 0,096 | 0,1020 < 0,78 |
